# Emotion management and stereotypes about emotions among male nurses: a qualitative study

**DOI:** 10.1186/s12912-021-00641-z

**Published:** 2021-06-29

**Authors:** Sergio Martínez-Morato, Maria Feijoo-Cid, Paola Galbany-Estragués, Maria Isabel Fernández-Cano, Antonia Arreciado Marañón

**Affiliations:** 1 CAP Vila Olímpica, Parc Sanitari Pere Virgili, Barcelona, Spain; 2 Department of Nursing. Faculty of Medicine, Universitat Autònoma de Barcelona, Bellaterra, Spain; 3 Grup de Recerca Multidisciplinar en Salut i Societat (GREMSAS), (2017 SGR 917), Barcelona, Spain; 4 Research Group on Methodology, Methods, Models and Outcomes of Health and Social Sciences (M3O), Faculty of Health Science and Welfare, Centre for Health and Social Care Research (CESS), University of Vic‐Central University of Catalonia (UVIC‐UCC), 08500 Vic, Spain; 5 AFIN Research Group and Outreach Centre, Autonomous University of Barcelona Campus Bellaterra, Carrer Vila Puig, s/n, Edifici B-13, 08193 Cerdanyola del Vallès, Spain

**Keywords:** Emotion management, Male nurses, Gender roles, Gender stereotypes

## Abstract

**Background:**

Nursing requires a high load of emotional labour. The link between nursing, emotional labour and the female sex, complicates the figure of the male nurse, because masculinity is associated with physical or technical (rather than emotional) and moreover is defined in contrast to femininity. Our objective was to understand how emotion management is described by male nurses who work in the paediatrics department of a Spanish tertiary hospital.

**Methods:**

Qualitative descriptive study. The participants were selected through intentional sampling in the paediatrics department of a Spanish tertiary hospital. We conducted semi-structured interviews until reaching data saturation. We carried out a content analysis, using Lincoln and Guba’s definition of scientific rigour.

**Results:**

We identified two key themes in the data: 1) Stereotypes related to the emotional aspects of care: Participants took for granted some gender stereotypes while questioning others and defended alternative ways of managing emotions related to care. 2) Emotion management strategies: Participants described keeping an emotional distance, setting boundaries, relativising problems and using distraction and humour.

**Discussion:**

Nursing care is conditioned by gender roles and stereotypes that present men as less capable than women of feeling and managing emotions. However, emotion management is necessary in nursing care—especially in paediatrics—and our participants reported using strategies for it. Although participants continued to interpret care in terms of traditional roles, they contradicted them in adapting to the emotional labour that their job requires.

**Conclusions:**

New behaviours are emerging among male nurses, in which care and emotion management are not exclusively the purview of women. Our participants reproduced some gender stereotypes while disrupting others, and they tended to cling to the stereotypes that were favourable to them as male nurses. As we work towards a gender-neutral profession, these results represent a first step: male participants reported that they provide care and manage their emotions as well as (or better than) women. However, because they substantiated their claims by drawing on negative stereotypes of women, further progress must be made.

## Introduction

The presence of men in nursing is significantly lower than that of women. According to the Spanish National Institute of Statistics [1], for the last 10 years, about 15% of Spain’s nurses have been men, and this proportion is increasing. Internationally, the figures range from 2% in China (2017) [2] and 7% in Canada (2016) [3] to 9.6% in the US (2018), 10.5% in Sweden (2016) [4], 11% in the United Kingdom (2017) [5], and 11.7% in Australia (2018) [6]. According to the World Health Organisation, in 2019 the proportion of male nurses was 14% in Europe and the Americas, 19% in Oceania, 21% in Asia and 35% in Africa [7].

Nursing has traditionally been carried out by and associated with women; as a result, men who wish to become nurses often find that their masculinity is questioned by others [8, 9]. Professions traditionally practiced by women are associated with a higher load of emotional labour [10]. According to Hochschild [11], norms surrounding the expression of emotions vary cross-culturally. Emotions form part of daily life and allow us to understand motives underlying our own and others’ actions. Emotions reflect personal and collective affective states, and, because of their multi-faceted nature (biological, psychosocial and cultural), they play a determining role in the construction, maintenance and transformation of the social order. Emotional labour conducted in the workplace is often viewed as an inherent attribute of the profession in question. Professionals must deal with their own emotions without forgetting that their social context conditions how they manage them. Traditionally men are assigned a role in which emotion management is considered secondary, because it is associated primarily with women [12, 13].

Nurses are expected to show empathy, but, at the same time, detachment is identified as a trait of professionalism [14]. Using emotion management strategies makes it possible to satisfy one’s professional obligations [15, 16]. However, while having good emotion management strategies protects the individual [17], emotion management in itself involves stress, which appears to justify detachment and emotional disengagement to overcome suffering in the workplace [10]. In paediatric care, emotions are particularly important. Working with children means having to handle the emotions of children, their families and the professionals themselves. These emotions are linked to sickness and death in childhood and are subject to shared values and beliefs and individual experiences [18]. It is precisely in emotion management where nurses have the most difficulty [19, 20].. A meta-analyisis shows that paediatric nursing is the area in which burnout is most prevalent [21].. According to other studies, burnout is more prevalent in male nurses, both in paediatrics and other areas [18, 22].

Nursing is marked by the norms of social interaction that are linked to gender. As men, male nurses are often typecast in conventional masculinity, setting aside their emotional side to present themselves as rational, autonomous and technically or physically competent [23]. Men, regardless of their occupation, tend to use rational coping strategies—developing plans to manage problems, establishing realistic expectations—which is in turn linked to less chronic stress [12]. However, in the context of professional care, male nurses have to reformulate their role as men to adapt to the emotional nature of their work. For example, they use empathy to improve their therapeutic relationships and provide better care [24]. Some patients highlight experiences in which male nurses were respectful and considerate, used active listening and supported them. Importantly, these characterisations could result from a contrast between expectations and experiences of being cared for by a male nurse [25]. Male nurses alternately reject and reproduce hegemonic masculinity, which presents them as less able than women to provide emotional support [26]. This double-bind might be a factor contributing to the fact that male nurses are more likely to experience stress than female nurses [18, 22].

The traditional rigid definitions of masculinity—a reflection of patriarchal culture—compromise the masculinity of men who choose a profession that is “naturally” associated with women [27]. At the same time, these definitions call into question the acceptability of men in the profession [28]. This points out the necessity of analysing how male nurses manage the emotions linked to care and explore new non-hegemonic constructions of masculinity. Several researchers have investigated the figure of the male nurse, highlighting stereotypes and difficulties that male nurses find on the job [29, 30] but also the advantages of incorporating men into care work [2, 31]. Others have focused on the importance of emotion management in nursing in general [32, 33]. However, little attention has been paid to emotion management among male nurses in particular, despite the fact that care environments foster emotional development, and men are often associated with roles that make them feel greater discomfort in the work environment [25, 34, 35]. Exploring how men manage their emotions in professions considered to be women’s professions, such as nursing (a field that also carries a high emotional charge) will make it possible to advance knowledge about men’s emotion management strategies and the gender stereotypes that are manifested in the area of care.

This study is part of a multidisciplinary project about the participation of men in professional and non-professional care entitled “Men as caregivers: challenges and opportunities to reduce the gender gap and to face new care need.” Under the auspices of this project, we published an earlier qualitative article [36] about how male nurses manage relationships with the families of hospitalised children and how these are influenced by gender roles and stereotypes. We showed that male nurses interpreted the relationship with families in terms of traditional gender roles and stereotypes, despite rejecting the notion that nursing is a women’s profession. In the current article, we focus on a different question: How do male nurses describe emotion management on the job?

### Objective

Understand how emotion management is described by male nurses who work in the paediatrics department of a Spanish tertiary hospital.

## Methods

### Design

Descriptive qualitative study. We chose this design because it enabled us to collect information about how male nurses’ experienced emotions in paediatric care. It also provided rich descriptive content from the participants’ perspective [37].

### Setting and participants

The study population belongs to a public tertiary hospital in Barcelona, Spain, which employs over 3000 nurses. The maternal and child department treats an average of 300,000 patients per year and employs a total of 624 nurses, of which 31 are male (study population). To be included in the study, participants had to meet the following inclusion criteria: to be eager to participate; to be employed as a paediatric nurse in hospitalisation, emergency, or intensive care; and to have at least 1 year of experience working in these areas of paediatric care. The exclusion criterion was working in out-patient care, because such nurses do not have hospitalised patients.

Sampling was purposive [38] to ensure a both homogeneity-regularity and heterogeneity-diversity across the participants. In choosing participants, we considered age, work areas, professional experience and experience in paediatrics (Table 1). Consequently, we chose participants who had significant experience interacting with patients and families and who were motivated to speak with us. Data collection took place September–December 2015.

**Table 1:** Characteristics of the participants

Age (years)	25–60
Paediatric nursing experience	
1–10 years	7
11–30 years	5
Participants’ work area	
Paediatric Hospitalisation Unit	4
Neonatal Intensive Care Unit	3
Paediatric Intensive Care Unit	4
Paediatric Emergency Unit	1
Is the participant a parent?	
Yes	5
No	7

Supervisors were recruited to describe the project to male nurses in their unit and invite them to participate. The nurses who agreed to participate were screened by the principal investigator (PI) using the diversity criteria that we had established. Each selected participant was then contacted by the PI, who explained the study details, secured his consent in writing and arranged a time and date for the interview. One participant withdrew at this point. The researchers didn’t know the participants before the study began. Participants were not compensated.

### Data collection

The PI conducted semi-structured interviews [38] with the participants in Spanish, between September and December 2015. The vast majority of the interviews (11) were held at a university classroom before or after the nurse’s shift. One participant requested that the interview be held at a hospital office. We developed a semi-structured interview guide, making minor changes after the first interview. We asked: How do you feel when you work caring for hospitalised children and their families? Could you tell me about your emotions, in comparison to those of your female nursing colleagues, when you care for these children? What do you do to manage these emotions and how would you compare it to what female nurses do? We asked follow-up questions depending on how participants answered. Interviews lasted 50 min on average. The interviewer took field notes. We defined data saturation as the point at which interviewees repeated responses provided by previous participants, without providing new information [39]. We reached data saturation in interview 11. However, to be sure that saturation had been reached, we conducted a twelfth interview (which also showed repetition and the lack of new themes). We then ceased data collection. We audio recorded the interviews and later transcribed them. We did not return transcripts to participants. However, we provided them with a summary of their interview and allowed them to make changes, if necessary. All participants accepted their summary without changes.

### Data analysis

We carried out a content analysis of the data in the following steps. All recordings were transcribed verbatim. The PI and another author read and reread the transcripts and notes. Next, the PI coded the first four transcripts, which she later discussed with the other author until reaching consensus. From this point, these two authors divided the remaining transcripts between them, each coding some of them based on the shared criteria they had developed. They discussed any cases in which it was unclear how to apply the coding criteria. After the 12 transcripts were coded, they were shared with the remaining authors and analysis proceeded jointly in the team. Finally, the data were refined and grouped hierarchically into themes, and these themes were named [40]. We used the Dedoose software package to codify and organise the data.

### Rigour

We followed Lincoln and Guba in ensuring the credibility, transferability, dependability and confirmability of the study [41]. Credibility: Each participant was offered the chance to correct the summary of his interview. As mentioned, we conducted the initial data analysis and coding separately. Finally, we used the technique of constant review to compare our results with those reported in the scientific record. Dependability: We carried out constant review and a detailed description of how we collected, analysed and interpreted the data. Transferability: We collected data exhaustively and provided a close description of contextual factors and participants to allow comparison and potential extrapolatability to other contexts. Confirmability: Constant reflection encouraged us to consider our own positionality.

## Results

We interviewed 12 male nurses working in paediatrics. They ranged in age from 25 to 60. Participants’ characteristics can be seen in Table 1.

We identified two main themes in the interview data: stereotypes in the emotional aspects of care and emotion management strategies (Table 2). Stereotypes in the emotional aspects of care.

**Table 2:** Table of themes, categories and codes

**Theme**	***Stereotypes in the emotional aspects of care***			
**Codes**	▪ Nursing is a women’s profession ▪ Care is associated with females ▪ Comfort can be offered in different ways ▪ Innate characteristics of women: sweetness, the use of touch in care ▪ Naturally acquired maternal role ▪ More relaxed male nurses’ attitude in the therapeutic relationship ▪ Male nurses show their emotional needs less			
**Theme**	***Emotion management strategies***			
**Category**	Emotional distancing	Setting boundaries	Relativising problems	Using distraction and humour
**Codes**	▪ Not feeling part of the work environment ▪ Getting involved is harmful ▪ Getting involved is unprofessional	▪ Learning affective touch ▪ Not taking work home ▪ Not creating personal ties with patients/family	▪ Minimising the importance of work problems ▪ To relativise is to protect oneself emotionally ▪ Men relativise better than women	▪ Humour facilitates therapeutic relationship ▪ Play and distraction are useful in caring for children ▪ Greater sense of humour ▪ Greater use of play and distraction

The participants reported that providing care led them to face emotional challenges on a daily basis. They reported butting up against societal norms according to which care falls under the purview of women. For some participants, being a man in a women’s profession wasn’t easy, because the patient or the family usually expected to be attended by a woman. These situations reinforced the stereotype that nursing is a women’s profession and caused the male nurse to feel he wasn’t meeting the expectations of the patient and the family.

*“You may find a little more misunderstanding among people, (...) who don’t understand that a man might have to provide care for a woman.” (I1).*

Following this argument, participants believed that in the collective imagination care is still associated with the female figure, especially in the case of children or in situations that involve providing emotional care through the use of touch. Still, some participants questioned this hegemony and aimed to undermine this prejudice. When talking about a female nurse he used to work with, one participant reported:

*“‘A man can’t do this job well. A man can’t bathe a child, take care of a child, show affection toward a child. A man can’t do it well.’ That was her argument. But then I set myself a goal and it was to show her that we could do well” (I8).*

Participants also reported that they had developed their own ways of handling the emotional aspects of caring for children. According to them, emotional and physical comfort can be offered in different ways by male nurses.

*“Well, you can comfort him [the child] in many other ways: you can use touch differently, you can hold them in your arms and calm them in your arms, there are many ways. Just like women don’t all show affection in the same way, do they? That’s what I think.” (I8).*

However, the participants attribute to women supposedly innate characteristics such as sweetness and the use of touch in care, as well as a naturally acquired maternal role. Additionally, they described the characteristics that they considered to be feminine to facilitate the therapeutic relationship. They described these attributes as complex, in contrast to the emotional simplicity with which most of them identified.

*“Because women may be more tactful when it comes to... men are like simpler, I think.” (I12).*

*“You have that component of sweetness and you can reach [patients] more easily with that component of sweetness, of a sweeter approach, closer to families in specific situations. And guys, well, we may keep a little more distance in that regard.” (I3).*

While participants highlighted these characteristics, they also associated female nurses’ maternal behaviour with authoritarianism. In contrast, according to them, male nurses have a more relaxed attitude in the therapeutic relationship.

*“Well, that facet, on the positive site, my previous [female] teammate was more like a mother: “You have to take it because of this and that (...)” But I, for example, break protocol a little bit. Maybe I picked up the child, put on music for her, danced with her... We stimulated her in another way.” (I3).*

One of the participants asserted that some men possess a more developed maternal feeling than others. This trait would in turn lead them to want to devote themselves to professional care.

*“This depends on the person. You have to like it [providing care]. (...). Maybe some of us men that have more maternal feeling.” (I7).*

According to the participants, male nurses show their emotional needs less and, in fact, do not express them in the workplace. Women, on the other hand, show more of their sensitivity, affecting, according to them, their ability to perform their job.

*“In the general emergency room and in traumatology a male co-worker has never told me, “Man, take over for me. I can’t do it because I feel like crying” or “because I can’t cope.” Because it’s never happened to me with a male co-worker, but it has happened to me with a female co-worker.” (I5).*

### Emotion management strategies

When children are hospitalised, emotions run strong and nurses are forced to respond. Although participants described themselves as less emotional and more objective because they were men, they also claimed that being male did not prevent them from expressing and managing emotions.

*“The first death that I had in paediatrics, I wanted to keep working. ( … ) Then I left the room and I started crying. I needed a quarter of an hour. And actually, now that I’m telling you about it, I’m getting emotion, you know (...) because we get more emotional with a child” (I5).*

In our analysis, we detected four strategies that the participants used to manage their emotions in the care environment: emotional distancing, setting boundaries, relativising problems and using distraction and humour.

#### Emotional distancing

According to participants, the cultural expectations that attribute to women the caring role leads many male nurses to distance themselves emotionally when faced with questions about how the patient and families will react to a male nurse.

*“At the beginning, I think there’s a difference in behaviour for the good, because you don’t know how they’re going to accept a male nurse.” (I2).*

The participants reported that they become less involved emotionally than their female co-workers. For some of them, as expressed by I1, getting too involved isn’t healthy and makes it hard to be professional.

*“You establish a professional relationship, polite. But you don’t get to the point of getting involved emotionally (...). We don’t get involved at an emotional level the way a woman could.” (I12).*

*Sometimes you get really involved (...) and I think it’s not healthy (...). It’s very difficult sometimes to be professional when you have very mixed feelings for a certain patient.” (I1).*

#### Setting boundaries

For these participants, certain activities, such as providing affectionate touch, are not part of the profession, but they were forced to learn them because this was expected of them in paediatrics. Emotions linked to affectionate touch don’t go beyond the professional context. This delimitation is identified as an attribute of professionalism that supports the child’s health.

*Yes, we can provide emotional and physical connection, but it’s not our role. We do it like someone who’s learned to play a game and practices it (...). You can get into it, like you’re acting, but then you don’t take that problem home. We’re here to provide the most professional care possible, and definitely the most humane possible. But also professional. And when we mix feelings, we stop being professionals.” (I1).*

Participants reported that, on occasion, their female co-workers crossed the lined in the therapeutic relationship, creating attachments outside of work with the people they cared for. According to participants, this doesn’t occur with male nurses.

*“Maybe being friends or palling around can take place sometimes. This, in the case of my male co-workers and me, doesn’t happen.” (I12).*

#### Relativising problems

Relativising means downplaying the importance of problems that emerge on the job. This strategy can minimise the effect on the nurse and also bring about a simple resolution to problems. This supposedly different approach that men and women use to handle their professional lives is also reproduced in personal life. In the words of one participant, men and women have *“different ways of taking things in life” (I8).*

*“When we [men] have a problem that’s pretty serious and can affect us even in our partner relationship, we have a tendency to relativise it, to play it down. I think women don’t relativise as much.” (I5).*

Participants reported that relativising problems that emerge around care was healthier and led to a greater emotional protection. According to them, women don’t relativise as much as they do, and they experience these problems more personally, which leads to greater worry and reduces their well-being.

*“Women take everything more personally, you know? And they experience it more like a pressing problem, they find themselves more worried, more anxious.” (I8).*

#### Use of distraction and humour

Distraction and humour helped participants manage situations that were emotionally problematic. They described this emotional strategy as being more typical of men and said that it facilitated the therapeutic relationship and helped them handle moments of tension surrounding their patients’ health. According to the participants, women handled these situations more emotionally, while they tended to use other strategies, such as joking around.

*“I try to make jokes to release some of the tension that the problem has caused (...). I do this more with the parents (...) I mean, you use what could be a problem as something positive, to downplay it and to get them to calm down. (...) What my female co-workers achieve with emotion, I achieve with jokes.” (I5).*

The use of games was also identified by participants as necessary when they treated paediatric patients. They reported that playing games helped them get closer to the child in the care environment and helped avoid complications in carrying out their work.

*“With a glove, I blow it up and I make a drawing or a face. Through games I get quite close to the child. Then I see that I can get closer to him (...) If you move in with a serious face, we’re on the wrong track (...). The child is going to start crying and everything is going to be more complicated (...). You always have to try to have a smile on your face” (I7).*

As we can see, when participants talked about what supposedly distinguished them from female nurses, they relied on positive stereotypes attributed to the male sex, such as knowing how to keep emotional distance and set boundaries, relativising problems and having a better sense of humour.

## Discussion

The participants identified themselves as less emotional in care environments than female nurses and reported expressing their emotions differently than their female counterparts, although they also argued that male nurses express emotions. Previous studies suggest that similar findings are the result of social expectations [25] and that male nurses masculinise care to protect themselves from exhaustion and emotional overload [42]. The social perception that emotional expression is necessary for nurses and that it comes naturally to women is at the core of the idea that nursing is a women’s profession [43]. These cultural associations/social perceptions mean that men engaged in professional care face a series of expectations and prejudices.

Popular representations of Florence Nightingale led to the recognition of women in professional care, the exaltation of women as ideal carers and the idea that nursing is a women’s profession [8].. Social expectations often condition the preferences of patients—leading them to prefer being cared for by a woman. This means that men must adapt their behaviour for a more effective therapeutic relationship [24, 44]. Other researchers have shown that men often face situations in which they feel excluded in care situations [35, 45], although slowly the figure of the male nurse is becoming consolidated and accepted in professional care [30]. Our participants also felt excluded because they were men. Men and women share the same motive for becoming nurses: being able to care for other people [46]. Further, the main reasons that some men choose not to become nurses or leave nursing are related to stereotypes surrounding the profession [9]. In fact, some participants also explained that some men develop a “maternal feeling” that leads them to devote themselves to professional care. Using the descriptor “maternal feeling” links nursing implicitly to a supposed feminine instinct. In terms of patients’ perspectives, some researchers show that patients highlight traits of male nurses that make them apt for providing care. However, these patients in fact identify the same traits in female nurses [25]. This set of findings reinforces the need to normalise nursing as a gender-neutral profession [26, 47].

Despite the fact that our participants claimed that their desire to provide care was a key factor in deciding to become nurses and despite the fact that they developed emotion management strategies, our participants interpreted care in terms of traditional gender roles when they said, for example, that they become less emotionally involved than their female co-workers. This discursive move highlights a simultaneous reproduction and disruption of the traditional, binary gender system, as seen in a previous article [36]. The study participants not only felt able to provide care, but also reported being able to develop emotional care strategies associated with femininity, when the situation required it. Notably, they defined themselves as “good care providers”, highlighting technical aspects about controlling situations or using physical force, to the detriment of emotional aspects of care. We observed that the participants clung to or rejected gender stereotypes depending on whether the stereotype was favourable to them in their profession. Other researchers show that continually having to decide whether to act according to or against normative masculinity causes psychological stress, meaning that gendered expectations condition men’s well-being [48].

Integrating a focus on gender into the study of men who do “women’s work” highlights the need for research to be oriented toward multiple masculinities instead of a single mode [27, 49]. For the men in our study, emotions linked to care are something to be learned and they depend on the care context. Paediatric care often requires the management of difficult emotions that emerge from stressful situations related to communication, suffering and taboos in care for children [18, 50]. The literature highlights that male nurses in paediatric care are more likely to suffer from stress and burnout [21, 22, 50]. The data show that participants developed emotional practices when necessary, depending on the context, as also described in an earlier study [49]. When male nurses express a preference for more technical areas, this preference can in itself represent a strategy of emotion management, since it makes it possible to feel a sense of accomplishment in a scenario in which the probability of success (such as the survival of the person being cared for) can be low [24]. Notably, overall distress is more prevalent in women regardless of occupation, and women are more likely to use emotional coping strategies [12]. However, in the case of nursing, distress is more prevalent in men and men use rational coping strategies [18, 22].

The first strategy we detected was **emotional distancing**. This strategy involves staying emotionally separate from patients to maintain neutrality, an approach that is also described as feeling separate from events and the emotions that they generate [12], which is identified with greater protection of the nurse’s mental health and a more professional treatment of the patient [51]. Emotional distancing, in the more physical sense of the term, is associated with males [10]. Physical contact is conditioned by gender in the daily life of the male nurse, as a kind of taboo [31]. In fact, this taboo leads some male nurses to choose to work in areas of care in which physical contact is less necessary [34], highlighting how stereotypes affect emotion management strategies and the choice of care area.

Harris argues that inflexible distancing establishes hierarchies of power among professionals and the people they care for, generating asymmetrical therapeutic relationships [52]. Although emotional distancing is recognised in the literature as typical of men, it is a strategy that is employed by nurses of both sexes [15]. In addition, as described by some study participants, this emotional distancing emerges from feeling insecure about how they will be received as men in a feminised profession. However, emotional distance is described as a mental health strategy, in that it prevents the patients’ pain and negative feelings from contaminating the emotional labour of the nurse, thus helping to maintain professionalism [51].. Our participants described this strategy as one used by men and linked it to greater professionalism. Notably, prior research points to the importance of finding a balance between involvement and distance, to avoid being negligent in the provision of care [53].

The participants defined **setting limits** as being able to feel and to carry out the emotional labour required of the profession without taking these emotions beyond the work environment. The participants described establishing limits as a strategy that helped them strike a balance between respecting the patient’s privacy and understanding his or her emotional world through the therapeutic relationship, which improves the satisfaction of both the patient and the nurse [15]. A therapeutic relationship in which emotional limits are balanced increases satisfaction and reduces stress in palliative care for paediatric patients [53]. Hammonds et al. [14] show that nurses (without reference to sex) typically set boundaries outside of work but also report that these limits are not uncrossable.

Our participants talked about facing day-to-day problems in their jobs by **relativising** them. Similarly, Lartey et al. [32] show that nurses use strategies to minimise the importance of things that happen on the job, normalising their emotional experiences to cushion the impact of the emotional demands of professional caregiving. In our study, participants took this minimisation beyond the professional environment, identifying it as a natural attribute of men. Other studies, in contrast, describe this strategy as intrinsic to nursing [33]. This contrast again points out the tendency of our participants to interpret care in terms of traditional gender roles.

Participants talked about using **distraction and humour** as a tool to reassure and comfort the people they cared for and their families. Strategies designed to provide comfort themselves act as a protective factor for nurses who use them, and these strategies are associated with better psychological well-being, happiness and satisfaction [24, 54]. Humour additionally serves to humanise health care and therapeutic communication, which requires practice [55]. Other studies do not make gender-based distinctions in this area, while our participants again understood this strategy to be the purview of men. Here, too, they espoused a gender stereotype that benefited them.

This study has several limitations. First, data collection took place at a single centre. However, the centre had a large number of both male and female nurses, a plurality of care department and high complexity of care. A second limitation is that we didn’t interview female nurses, meaning that we couldn’t compare perceptions of male nurses with those of female nurses. Third, the interviews were conducted by a woman, and the responses of participants could have been conditioned by the gender difference between them. In any case, the interviewer’s sex did not prevent participants from reproducing negative stereotypes about women, leading us to think that any effect of the interviewer’s sex was small. Additionally, our team includes a male paediatric nurse, who was able to contribute his point of view to the analysis. Finally, we did not complement our interviews with direct observation in the hospital, which would have allowed us to collect empirical data about male and female nurses’ emotional behaviour for comparison with the interview data. Nonetheless, the study gives voice to male nurses, allowing us to understand how they view emotion management strategies and how they grapple with gender stereotypes as they forge a new masculinity.

## Conclusions

Emotion management is necessary for professional carers, especially those in paediatrics, and male nurses learn strategies for it. Professional care is conditioned by traditional gender roles, in which men are understood as less capable of feeling and managing emotions. However, as human beings, both male and female nurses manage their emotions; in no sense is emotion management the exclusive terrain of women. Nonetheless, our participants tended to cling to gender stereotypes that favoured them in their professional role, including negative stereotypes of female nurses as over-emotional on the one hand and humourless on the other. Future research should explore other factors that influence the gender stereotypes linked to care, as well as the link between gender stereotypes and stress on the job. It is also important to explore how female nurses and the general population perceive male nurses, especially in relation to the emotional aspects of care. Finally, it is important to learn whether female nurses use emotion management strategies linked to masculinity, both in paediatric nursing and other areas.

Further work needs to be done to develop nursing as a gender-neutral profession, based on a definition of care as a task of all humans rather than only of women. Taking these steps would both give nurses—male and female—the professional recognition they deserve and open the door to more men to join the nursing profession, addressing the international shortage of nurses. Making these changes requires handling questions related to gender in undergraduate nursing education and in subsequent training.

## Data Availability

The datasets used and/or analysed during the current study are available from the corresponding author on reasonable request.

## Abbreviations

PI: Principal Investigator
I: Interview
